# Noise-immune zeroing neural dynamics for dynamic signal source localization system and robotic applications in the presence of noise

**DOI:** 10.3389/fnbot.2025.1546731

**Published:** 2025-02-05

**Authors:** Yuxin Zhao, Jiahao Wu, Mianjie Zheng

**Affiliations:** ^1^School of Humanities, University of Westminster, London, United Kingdom; ^2^School of Information and Intelligent Engineering, Guangzhou Xinhua University, Guangzhou, Guangdong, China; ^3^School of Computer Science and Software Engineering, Shenzhen University, Shenzhen, China

**Keywords:** dynamic signal source localization, robotic manipulator, angle-of-arrival (AoA) scheme, time-difference-of-arrival (TDOA) scheme, trajectory tracking scheme, noise-immune zeroing neural dynamics (NIZND)

## Abstract

Time angle of arrival (AoA) and time difference of arrival (TDOA) are two widely used methods for solving dynamic signal source localization (DSSL) problems, where the position of a moving target is determined by measuring the angle and time difference of the signal's arrival, respectively. In robotic manipulator applications, accurate and real-time joint information is crucial for tasks such as trajectory tracking and visual servoing. However, signal propagation and acquisition are susceptible to noise interference, which poses challenges for real-time systems. To address this issue, a noise-immune zeroing neural dynamics (NIZND) model is proposed. The NIZND model is a brain-inspired algorithm that incorporates an integral term and an activation function into the traditional zeroing neural dynamics (ZND) model, designed to effectively mitigate noise interference during localization tasks. Theoretical analysis confirms that the proposed NIZND model exhibits global convergence and high precision under noisy conditions. Simulation experiments demonstrate the robustness and effectiveness of the NIZND model in comparison to traditional DSSL-solving schemes and in a trajectory tracking scheme for robotic manipulators. The NIZND model offers a promising solution to the challenge of accurate localization in noisy environments, ensuring both high precision and effective noise suppression. The experimental results highlight its superiority in real-time applications where noise interference is prevalent.

## 1 Introduction

Generally, in a dynamic signal source localization system, a sensor array is deployed within a reasonable range. This array is used to measure real-time dynamic variables, such as time of arrival differences and arrival angles, from a dynamic target object to individual sensors. Subsequently, a mathematical model is established based on the mobile target object's position and the real-time dynamic variables acquired by the sensors. Finally, an appropriate solving method is employed to achieve real-time solutions, obtaining the accurate real-time position of the dynamic signal source.

The solving of dynamic signal source localization (DSSL) problems continues to be utilized in numerous scientific computing and engineering applications. In recent years, an increasing number of scholars have started to work on this class of problems. Such problems play a crucial role in a wide range of applications such as quadrotor positioning (Zhao et al., 2021), robotics (Xie et al., 2024b; Sun et al., 2024), smart furniture (Nassar et al., 2019), mine personnel operations (Zare et al., 2021), and so on (Jin et al., 2024a; Liu et al., 2024, 2022; Wu et al., 2023). However, an overview of real-life production shows that dynamic source location tracking has not been effectively applied in areas where it seems to be urgently needed. For example, in the case of mega-malls (Ali et al., 2019), where satellite coverage is not available but positioning is urgently required, dynamic source location tracking combined with 3D positioning solutions can be used to achieve indoor positioning and thus enable navigation indoors (Guo et al., 2019a; Kunhoth et al., 2020). From a lifestyle perspective, this is much more convenient and practical.

Various source location solutions have proven effective and can be broadly classified into the following three categories. (1) Time of arrival (TOA) scheme, where localization is achieved by measuring the time it takes for a signal emitted from a source to reach several different location sensors. (2) Time of Difference of Arrival (TDOA) scheme, where positioning is achieved by measuring the difference between the time of arrival of the signal from the source at the main sensor and the time of arrival of the signal at each of the other different position sensors, respectively, and then calculating the positioning. (3) Angle of Arrival (AoA) scheme, where positioning is achieved by measuring the angle of arrival of the signal emitted from the source to each of the sensors at different locations (Guo et al., 2019b; Wu et al., 2019a).

The TOA scheme is one of the most common schemes of location. For example, Guo et al. (2019b) proposed a self-clustering measurement combination scheme. The scheme deals with the unclear relationship between the TOA measurement data and signal source in the multi-source location problems. Wu et al. (2019a) introduced synchronization errors into the TOA scheme based on the no-line-of-sight (NLOS) positioning model, which improves the positioning accuracy in NLOS propagation. Besides, to further tolerate measurement errors, NLOS errors, and synchronization errors, Wu et al. (2019b) proposed two new artificial neural network localization schemes and a TOA measurement scheme. As an improvement of the TOA scheme, the TDOA scheme has been the subject of many positioning studies. For instance, Wu P. et al. (2019) proposed a hybrid firefly algorithm (Hybrid FA) scheme that combines the weighted least squares algorithm and FA. This scheme reduces the calculation amount of common passive positioning methods and improves the positioning accuracy (Wu P. et al., 2019). Wang et al. (2020) proposed a TDOA estimation based on Kronecker product decomposition, which applies to effectively identify the relative acoustic impulse response between two microphones. To be suitable for short-distance positioning problems, Pérez-Solano et al. (2020) proposed a UWB indoor positioning system based on the TDOA scheme. To date, various methods have been developed and introduced to improve the AoA scheme. Monfared et al. (2020) used the Non-Data-Aided iterative algorithm to iterate between angle and position estimation steps to gradually improve the AoA positioning accuracy. Then, Monfared et al. (2021) improved the performance of the AoA scheme by comparing the variance of the middle estimated position of different combinations of all possible anchor point sets with pre-calculated thresholds. In addition, Zhou et al. (2022) proposed an angular domain AoA estimation scheme to locate the user. Another, Hong et al. (2020) studied the AoA positioning in visible light and improved the accuracy of the AoA positioning based on the quadrant-solar-cell and third-order ridge regression machine learning algorithm.

All three of these source localization schemes have their advantages and disadvantages. However, the TOA scheme is simple and has low complexity, but since TOA uses the transmission time of the base station and the target to be measured to calculate the transmission distance to further determine the location of the tag, which requires clock synchronization between each base station and the target to be measured, TOA is susceptible to clock interference in complex indoor environments, resulting in serious errors in positioning accuracy. TDOA scheme, compared with the TOA scheme, does not need to keep the clock synchronization between each base station and the target to be measured, but only needs to synchronize between base stations. This makes the TDOA scheme easier to implement and its application is broader. Compared with the other two schemes, the AoA scheme is suitable for positioning at shorter distances and is generally used as an auxiliary tool for primary coarse positioning (Jin et al., 2016; Ferreira et al., 2005; Jiang and Wang, 2003).

Robotic manipulators have gained significant attention in recent years and have been employed across various fields (Xie et al., 2024a; Jin et al., 2024c; Sun et al., 2003). The trajectory tracking of robotic manipulators is a crucial topic in robotic investigation (Jin et al., 2024b; Xie and Jin, 2023; Lian et al., 2024). In Zhai and Xu (2020), a singularity avoiding sliding mode control was presented, achieving trajectory tacking for robotic manipulators. By leveraging vector pseudo distance, Yang et al. (2022) developed an obstacle avoidance control method for a redundant manipulator, which outperforms traditional methods using Euclidean distance. A learnable motion control strategy was designed in Xu et al. (2020). Through utilizing position and velocity information, it can address the parameter adjusting problem in the controller.

The recurrent neural dynamic (RND) model is often considered a classical intelligent computational approach and has been extensively studied in many scientific and engineering fields (Li et al., 2019a; Zhang et al., 2018; Li et al., 2019b). Neural dynamics transmits and updates information through neurons, representing a brain-inspired algorithm. On this basis, a traditional gradient-based RND model (TGND) was proposed by Xiao et al. (2019) to deal with time-varying matrix inversion problems. However, Jin et al. (2022) pointed out that the TGND model could not make effective use of the time derivative information in solving time-varying problems, and the obtained state solutions would generate a time lag error. As the time lag error can seriously hinder the solution of dynamic localization problems. Therefore, traditional zeroing neural dynamic (TZND) (Dai et al., 2024), named after their inventors, were considered as a way to effectively utilize time derivatives to time-varying problems. Researchers have then continued to explore and investigate and devise various improved ZND models to solve different time-varying problems, including the application of activation functions. In Yan et al. (2021), A TZND model was proposed to solve a receding horizon control scheme for a redundant manipulator.

**Figure 1:**
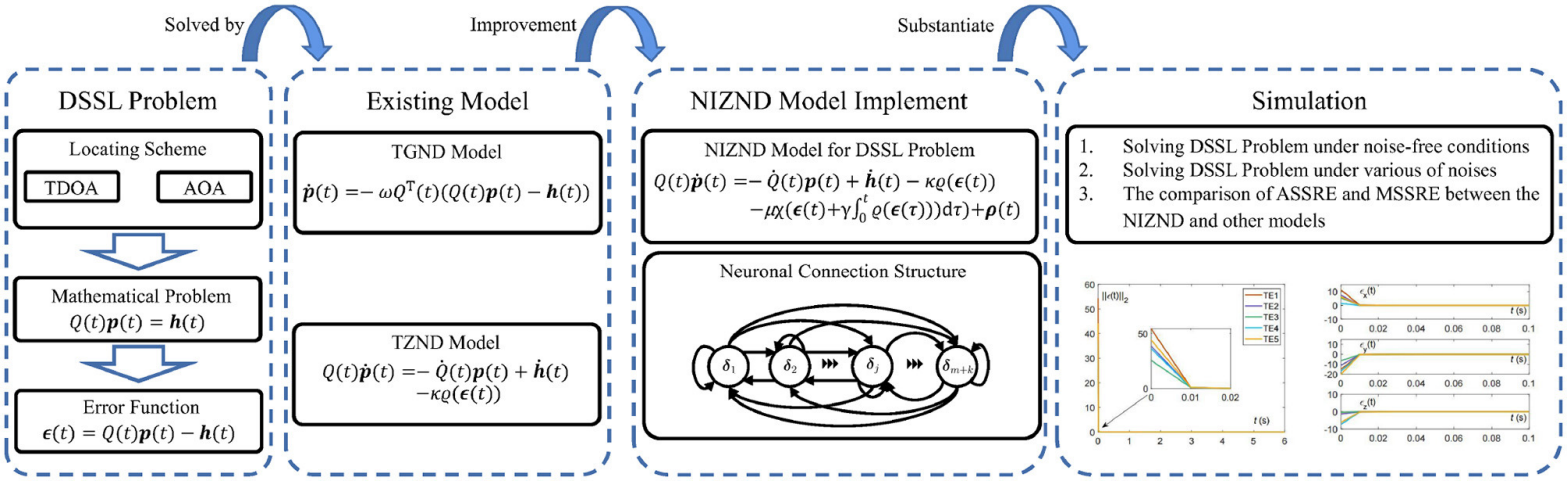
Block diagram of the structure of this paper.

On the one hand, as the dimensionality of solving dynamic localization problems and the degrees of freedom of robotic manipulator increases, the currently available methodologies are constructed to have low accuracy and long solution times when dealing with this type of problem. On the other hand, noise interference, as an important factor affecting accuracy, should further enhance the robustness and usability of the existing TZND model to achieve facing various noise disturbances brought about by realistic environments. To this end, a Noise-Immune Zeroing Neural Dynamics (NIZND) model activated by SBP is proposed in this paper to solve the DSSL problem and trajectory tracking scheme under noisy interference conditions. In the ideal environment without noise, the error of this model converges globally to zero; in the conditions of constant and random noise, the proposed NIZND model converges globally to a bounded range. To visually explain the design framework idea of this paper, a graphical representation of structure of this paper is shown in Figure 1.

The remainder of this article is arranged as follows. In Section 2, the DSSL problem is presented and transformed by both TDOA and AoA methods. Next, the specific design procedure of the proposed NIZND model is presented in Section 3, which also presents the derivation of the subsequent simulation part of the comparison models. Then, the corresponding analyses and proofs of the global convergence and robustness of the proposed NIZND model are presented in Section 4. After that, Section 5 provides several sets of illustrative simulation experiments that verify the high accuracy as well as the strong robustness of the NIZND model in its application to TDOA, AoA, and trajectory tracking schemes. Finally, the conclusion is summarized in Section 6.

Before ending the introduction, the main contribution of this paper is listed as follows:

- In this paper, a novel NIZND model is proposed to solve the DSSL and trajectory tracking problem of robotics, which has higher accuracy solution results and faster convergence speed in the iterative process than the traditional ZND model.
- A special activation function termed the SBP function in the real-valued domain is presented for constructing the NIZND model. Furthermore, this paper analyzes the anti-noise performance of the framework under different noise conditions and compares the performance of other models through theorems and proofs.
- Corresponding experimental results are executed for the DSSL problem and robotic problem, and the extraordinary superiority of the NIZND model is demonstrated by designing several sets of controlled simulation experiments.

## 2 Problem formulation and related work

Generally, the purpose of positioning technology is to set up base stations in a reasonable range, and the actual distance of the target object measured by base stations is calculated by the scheme to get an accurate target trajectory. In other words, utilizing the coordinates of base stations to measure the distance of the target object (Jin et al., 2020; Dai et al., 2021). Then, we introduce some essential definitions of the positioning schemes to model the geometric relationship between the target object and base stations into a time-varying dynamic matrix system.

### 2.1 Angle-of-arrival

**Figure 2:**
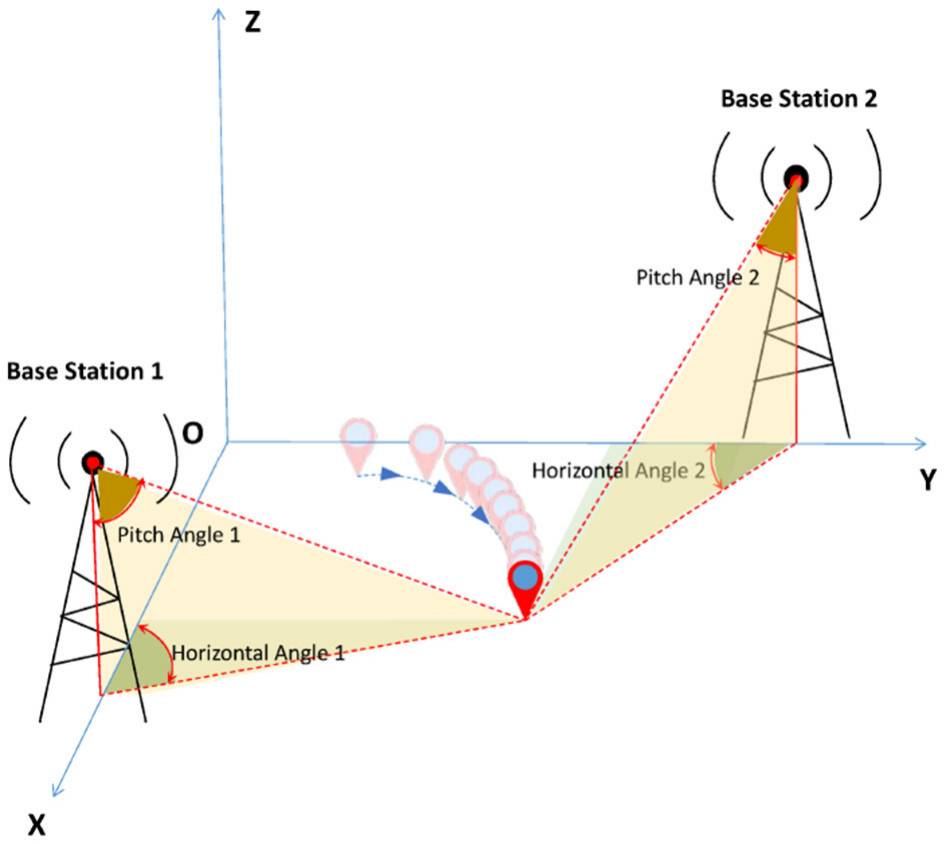
An illustration of the AoA scheme for dynamic signal source localization under two base stations.

The AoA scheme via measuring the horizontal and pitch angles between base stations and the target to calculate the intersection point. Then, according to the intersection point of direction line is formed between each base station and the target object to implement the positioning operation. For simplicity, Figure 2 illustrates the principle of the AoA scheme under two base stations. Note that the AoA scheme can be extended to a multi-base stations scheme, which enables to improvement of the performance of this scheme.

Suppose that the horizontal angles and the pitching angles of the *m* base stations are $\vec{\alpha }={\left[\alpha_{1}\left(t\right),\cdots \;,\alpha_{m}\left(t\right)\right]}^{\text{T}}\in {\mathbb{R}}^{m}$ and $\vec{\beta }={\left[\beta_{1}\left(t\right),\cdots \;,\beta_{m}\left(t\right)\right]}^{\text{T}}\in {\mathbb{R}}^{m}$, respectively. The superscript ^T^ indicates the transpose operation of a matrix or vector. The position of the target object at time *t* is expressed as $p\left(t\right)={\left[p_{x}\left(t\right),p_{y}\left(t\right),p_{z}\left(t\right)\right]}^{\text{T}}\in {\mathbb{R}}^{3}$, and coordinates of base stations are $S=\left[{\vec{s}}_{1},{\vec{s}}_{2},\cdots \;,{\vec{s}}_{m}\right]\in {\mathbb{R}}^{3\times m}$, where $s_{m}=\left[x_{m},y_{m},z_{m}\right]\in {\mathbb{R}}^{3}$ (Jin et al., 2020). According to the principle of AoA scheme, the geometric relationship between horizontal and pitch angle is formulated as


(1) $$\begin{array}{l} \alpha_{i}\left(t\right)=\text{tan}\left(\frac{p_{y}\left(t\right)-y_{i}}{p_{x}\left(t\right)-x_{i}}\right), \end{array}$$

(2) $$\begin{array}{l} \beta_{i}\left(t\right)=\text{tan}\left(\frac{p_{z}\left(t\right)-z_{i}}{\sqrt{{\left(p_{x}\left(t\right)-x_{i}\right)}^{2}+{\left(p_{y}\left(t\right)-y_{i}\right)}^{2}}}\right). \end{array}$$

We further define the dynamic matrix $Q_{1}\left(t\right)\in {\mathbb{R}}^{2m\times 3}$ and dynamic vector ${\vec{h}}_{1}\left(t\right)\in {\mathbb{R}}^{2m}$ as follows:


$$\begin{array}{l} Q_{1}\left(t\right)=\left[\begin{array}{ccc} {\vec{v}}_{1}\left(t\right) & {\vec{1}}_{m\times 1} & {\vec{0}}_{m\times 1}\\ {\vec{0}}_{m\times 1} & {\vec{v}}_{2}\left(t\right) & -{\vec{1}}_{m\times 1} \end{array}\right],{\vec{h}}_{1}\left(t\right)=\left[\begin{array}{c} {\vec{v}}_{3}\left(t\right)\\ {\vec{v}}_{4}\left(t\right) \end{array}\right], \end{array}$$

where vector ${\vec{v}}_{1}$, ${\vec{v}}_{2}$, ${\vec{v}}_{3}$, and ${\vec{v}}_{4}$ are constructed as


$$\begin{array}{c} {\vec{v}}_{1}\left(t\right)=\left[\begin{array}{c} -\tan\alpha_{1}\left(t\right)\\ \vdots \\ -\tan\alpha_{m}\left(t\right) \end{array}\right],{\vec{v}}_{2}\left(t\right)=\left[\begin{array}{c} \frac{\text{tan}\beta_{1}\left(t\right)}{\sin\alpha_{1}\left(t\right)}\\ \vdots \\ \frac{\text{tan}\beta_{m}\left(t\right)}{\sin\alpha_{m}\left(t\right)} \end{array}\right],\\ {\vec{v}}_{3}\left(t\right)=\left[\begin{array}{c} y_{1}-x_{1}\text{tan}\left(\alpha_{1}\left(t\right)\right)\\ \vdots \\ y_{m}-x_{m}\text{tan}\left(\alpha_{m}\left(t\right)\right) \end{array}\right],\\ {\vec{v}}_{4}\left(t\right)=\left[\begin{array}{c} \frac{y_{1}\text{tan}\beta_{1}\left(t\right)}{\text{sin}\alpha_{1}\left(t\right)}-z_{1}\\ \vdots \\ \frac{y_{m}\text{tan}\beta_{m}\left(t\right)}{\text{sin}\alpha_{m}\left(t\right)}-z_{m} \end{array}\right]. \end{array}$$

Therefore, the geometric relationship between the target object $\vec{p}\left(t\right)$ and base stations *S* is constructed as


(3) $$\begin{array}{l} Q_{1}\left(t\right)p\left(t\right)=h_{1}\left(t\right). \end{array}$$

### 2.2 Time-difference-of-arrival

TDOA is a time difference based localization scheme. The position of a target object is determined by measuring the difference in signal propagation time from the target object to multiple base stations and obtaining its distance difference (Dai et al., 2021).

Firstly, the purpose of TDOA is to utilize time differences to get dynamic distance differences between the target object and the base station. Such as distance differences:


(4) $$\begin{array}{l} \Delta l_{j}\left(t\right)=l_{j}\left(t\right)-l_{1}\left(t\right)=c\Delta g_{j}=c\left(g_{j}-g_{1}\right), \end{array}$$

where *j*∈{2, ⋯ , *n*}, symbol *c* represents the signal transmission speed. Δ*g*_*j*_ is the time difference between the arrival of the signal at the *j*th base station and the first base station. *g*_*j*_ is the time it takes for the signal from the target object to reach the *j*th base station. *l*_*j*_(*t*) denotes the dynamic distance between the target object and *j*th base station, and it satisfies:


(5) $$\begin{array}{l} l_{j}^{2}\left(t\right)\;=\;{\left(x_{j}-p_{x}\left(t\right)\right)}^{2}+{\left(y_{j}-p_{y}\left(t\right)\right)}^{2}+{\left(z_{j}-p_{z}\left(t\right)\right)}^{2}\\ \;=c_{j}-2\left(x_{j}p_{x}\left(t\right)+y_{j}p_{y}\left(t\right)+z_{j}p_{z}\left(t\right)\right)+p_{x}^{2}\left(t\right)+p_{y}^{2}\left(t\right)+p_{z}^{2}\left(t\right), \end{array}$$

where $c_{j}=x_{j}^{2}+y_{j}^{2}+z_{j}^{2}$. Subsequently, combining the Equations 4, 5, we get:


$$\begin{array}{l} \Delta l_{j}^{2}\left(t\right)+2l_{1}\left(t\right)\Delta l_{j}\left(t\right)=c_{j}-c_{1}-2\left(\Delta xp_{x}\left(t\right)+\Delta yp_{y}\left(t\right)+\Delta zp_{z}\left(t\right)\right), \end{array}$$

where Δ*x* = *x*_*j*_−*x*_1_, Δ*y* = *y*_*j*_−*y*_1_, Δ*z* = *z*_*j*_−*z*_1_. The following matrix *Q*_2_ and dynamic vector ${\vec{h}}_{2}\left(t\right)$ are furnished for further implementing the TDOA scheme (Equation 4):


$$\begin{array}{c} Q_{2}=\left[\begin{array}{ccc} x_{21} & y_{21} & z_{21}\\ x_{31} & y_{31} & z_{31}\\ \vdots & \vdots & \vdots \\ x_{n1} & y_{n1} & z_{n1} \end{array}\right]\in {\mathbb{R}}^{n\times 3},\\ {\vec{h}}_{2}\left(t\right)=\left[\begin{array}{c} \frac{1}{2}\left(c_{2}-c_{1}-\Delta l_{2}^{2}\left(t\right)\right)-\Delta l_{2}\left(t\right)l_{1}\left(t\right)\\ \frac{1}{2}\left(c_{3}-c_{1}-\Delta l_{3}^{2}\left(t\right)\right)-\Delta l_{3}\left(t\right)l_{1}\left(t\right)\\ \vdots \\ \frac{1}{2}\left(c_{n}-c_{1}-\Delta l_{n}^{2}\left(t\right)\right)-\Delta l_{n}\left(t\right)l_{1}\left(t\right) \end{array}\right]\in {\mathbb{R}}^{n}. \end{array}$$

Now, the TDOA scheme (Equation 4) is converted into the following as *Q*_2_(*t*)***p***(*t*) = ***h***_2_(*t*). In summary, the AoA (Equations 1, 2) and TDOA scheme (Equation 4) based DSSL can be transformed as the following dynamic linear equation problem:


(6) $$\begin{array}{l} Q\left(t\right)p\left(t\right)=h\left(t\right). \end{array}$$

## 3 NIZND model formulate

In this section, we construct the NIZND model to realize the AoA (Equations 1, 2) and TDOA scheme (Equation 4). To solve the DSSL problem (Equation 6) effectively, we first present the design process of the TGND model and the TZND model. Then, the second part designs the NIZND model with noise immunity based on the existing models.

### 3.1 Existing problem solver

The TGND model based on the gradient descent idea is often used to solve dynamic matrix system optimization problems (Tang and Zhang, 2023; Zhang et al., 2024; Lv et al., 2019). To monitor the performance of the model at any time, a performance metric called the error function is designed as


(7) $$\begin{array}{l} \epsilon \left(t\right)=Q\left(t\right)p\left(t\right)-h\left(t\right)\in {\mathbb{R}}^{n}. \end{array}$$

Then, a squared operation based on the error function can be written as $e\left(t\right)=||\epsilon \left(t\right)|{|}_{2}^{2}/2$. Moreover, according to the design philosophy of the TGND model (Xiao et al., 2020), we have:


(8) $$\begin{array}{l} -\frac{\partial e\left(t\right)}{\partial p\left(t\right)}=-Q^{\text{T}}\left(t\right)\epsilon \left(t\right). \end{array}$$

Finally, according to the above negative gradient descent information, one has:


(9) $$\begin{array}{l} \overset{.}{p}\left(t\right)=-\omega Q^{\text{T}}\left(t\right)\epsilon \left(t\right)\\ \;=-\omega Q^{\text{T}}\left(t\right)\left(Q\left(t\right)p\left(t\right)-h\left(t\right)\right), \end{array}$$

where the parameter ω>0 represents a scalar-valued factor used to control the convergence rate of TGND model (Equation 9).

As well as the TGND model (Equation 9), the TZND model is also a widely applied (Liao et al., 2024, 2022; Sun et al., 2022) and effective method for solving time-varying linear matrix systems (Equation 6). To begin with, the error function setting rules are the same as in equation (Equation 7). Then, the corresponding TZND model can be derived from the design formula:


(10) $$\begin{array}{l} \dot{\epsilon }\left(t\right)=-\kappa \varrho \left(\epsilon \left(t\right)\right), \end{array}$$

where κ>0 denotes a fixed parameter designed to control the speed of the solution process, and ϱ(·) denotes the scalar-oriented activation function. From the above equation, the following equation can be formulated as follow:


(11) $$\begin{array}{ll} Q\left(t\right)\dot{p}\left(t\right) & =-\dot{Q}\left(t\right)p\left(t\right)+\dot{h}\left(t\right)-\kappa \varrho \left(\epsilon \left(t\right)\right). \end{array}$$

### 3.2 NIZND model construction

The evolution function of the proposed NIZND model is formulated as


(12) $$\dot{\epsilon }(t)=-\gamma \varrho (\epsilon (t))-\mu \chi \left(\epsilon (t)+\gamma \int_{0}^{t}\varrho (\epsilon (\tau ))\text{d}\tau \right),$$

where γ>0∈ℝ and μ>0∈ℝ are the design parameters. Symbol χ(·) denotes the feedback-oriented activation function. In this paper, the following three activation functions are used to activate the model:

#### 3.2.1 Simplified activation function


$$\begin{array}{l} \varrho \left(\epsilon_{i}\right)=k*{\text{Lip}}^{\iota }\left(\epsilon_{i}\right). \end{array}$$

#### 3.2.2 Sign-bi-power function


$$\begin{array}{l} \varrho \left(\epsilon_{i}\right)=a_{1}*{\text{Lip}}^{\iota }\left(\epsilon_{i}\right)+a_{2}*{\text{Lip}}^{\frac{1}{\iota }}\left(\epsilon_{i}\right). \end{array}$$

#### 3.2.3 Combined activation function


$$\begin{array}{l} \varrho \left(\epsilon_{i}\right)=b_{1}{\text{Lip}}^{\iota }\left(\epsilon_{i}\right)+b_{2}\epsilon_{i}. \end{array}$$

Futhermore, the function Lip^ι^(·) can be defined as


$${\text{Lip}}^{\iota }\left(\epsilon_{i}\right)=\{\begin{array}{ll} |\epsilon_{i}{|}^{\iota }, & \epsilon_{i}>0,\\ 0, & \epsilon_{i}=0,\\ -|\epsilon_{i}{|}^{\iota }, & \epsilon_{i}<0, \end{array}$$

where ι>0. Consequently, it can be concluded that the proposed NIZND model with adaptive activation function for solving the DSSL problem (Equation 6) is written as


(13) $$\begin{array}{r} Q(t)\dot{p}(t)=-\dot{Q}(t)p(t)+\dot{h}(t)-\gamma \varrho (\epsilon (t))\\ -\mu \chi \left(\epsilon (t)+\gamma \int_{0}^{t}\varrho (\epsilon (\tau ))\text{d}\tau \right). \end{array}$$

Allowing for different noises interference, in order to further analyze and verify the influence of various noises on the NIZND model (Equation 13), the following form of the NIZND model (Equation 13) disordered by measurement noises is deemed:


(14) $$\begin{array}{l} Q(t)\dot{p}(t)=-\dot{Q}(t)p(t)+\dot{h}(t)-\gamma \varrho (\epsilon (t))\\ \;-\mu \chi \left(\epsilon (t)+\gamma \int_{0}^{t}\varrho (\epsilon (\tau ))\text{d}\tau \right)+\rho (t), \end{array}$$

where ***ρ***(*t*)∈ℝ^*n*^ is the inevitable noise during positioning. The system update is performed by ***p***(*t*) acting as neurons, and the proposed NIZND is a brain-inspired algorithm.

## 4 The theoretical analysis

In this section, the convergence of the NIZND model (Equation 13) to solve the DSSL problem (Equation 6) under ideal conditions and its robustness to different localization noises are demonstrated through theoretical analysis. Four theorems and corresponding proof procedures are summarized below.

### 4.1 Convergence

*Theorem 1:* Considering the DSSL problem (Equation 6) with non-noise perturbed, starting from any initial position within a certain range, the positional states generated by the proposed NIZND model (Equation 13) will converge to the theoretical position of the DSSL problem (Equation 6). That is to say, the residual error ||***ϵ***(*t*)||_2_ produced by the NIZND model (Equation 13) globally converges to zero.

*Proof:* First, a Lyapunov function is constructed for analyzing the convergence performance of the NIZND model (Equation 13):


(15) $$F_{1}(t)=\frac{\epsilon^{T}(t)\epsilon (t)}{2}\{\begin{array}{ll} >0, & \epsilon (t)\neq 0,\\ =0, & \epsilon (t)=0. \end{array}$$

Taking into account the subsequent proof process, let us define


(16) $$\begin{array}{l} \varsigma \left(t\right)=\epsilon \left(t\right)+\int_{0}^{t}\varrho \left(\epsilon \left(\tau \right)\right)\text{d}\tau . \end{array}$$

Thus, the derivative of ***ς***(*t*) with respect to *t* can be written as


(17) $$\begin{array}{l} \dot{\varsigma }\left(t\right)=\frac{\text{d}\varsigma \left(t\right)}{\text{d}t}=\dot{\epsilon }\left(t\right)+\varrho \left(\epsilon \left(t\right)\right). \end{array}$$

Without loss of generality, the fixed parameters in the NIZND model (Equation 13) are set as γ = μ = 1. Then, substituting Equation 13 into Equation 18, we can obtain:


(18) $$\begin{array}{l} \dot{\varsigma }\left(t\right)=-\text{Ψ}\left(\epsilon \left(t\right)\right). \end{array}$$

In the same principle as the construction of Equation 15, another Lyapunov equation is defined as


(19) $$F_{2}(t)=\frac{\varsigma^{T}(t)\varsigma (t)}{2}\{\begin{array}{ll} >0, & \varsigma (t)\neq 0,\\ =0, & \varsigma (t)=0. \end{array}$$

Consequently, the derivative of the Equation 19 is:


(20) $${\dot{F}}_{2}(t)=-\varsigma^{T}(t)\varrho (\epsilon (t))\{\begin{array}{ll} <0, & \varsigma (t)\neq 0,\\ =0, & \varsigma (t)=0. \end{array}$$

Since *F*_2_(*t*) is positive definite when ***ς***(*t*)≠0 and Ḟ_2_(*t*) is negative definite when ***ς***(*t*)≠0. Therefore, according to Lyapunov stability analysis, ***ς***(*t*) globally converges to zero. Furthermore, when ***ς***(*t*) = 0, it follows from the LaSalle's invariance principle that (Equation 18) can be reformulated as


(21) $$\begin{array}{l} \dot{\epsilon }\left(t\right)=-\varrho \left(\epsilon \left(t\right)\right). \end{array}$$

In light of the Equation 21, the time derivative of Equation 15 is expressed as


(22) $${\dot{F}}_{1}(t)=-\epsilon^{T}(t)\varrho (\epsilon (t))\{\begin{array}{ll} <0, & \epsilon (t)\neq 0,\\ =0, & \epsilon (t)=0. \end{array}$$

Similarly, Equations 15, 22 satisfy Lyapunov's second theorem, the error function ***ϵ***(*t*) can converge to zero globally. The proof is completed.

### 4.2 Robustness

*Theorem 2:* The residual error ||***ϵ***(*t*)||_2_ of the constant noise ***ρ***(*t*) = ***ρ***∈ℝ^*n*^ perturbed NIZND model (Equation 13) for solving the DSSL (Equation 6) globally converges to zero in the situation of −μχ(***ς***(*t*))+***ρ*** ≤ 0. The parameter ***ς***(*t*) represents the intermediate variable which is definition as Equation 16.

*Proof:* For the convenience of further derivation and analysis, the time derivative of intermediate variable ***ς***(*t*) with constant noise ***ρ*** is written as $\dot{\varsigma }\left(t\right)=-\mu \chi \left(\varsigma \left(t\right)\right)+\rho$, and its *i*th subelement is:


(23) $$\begin{array}{l} {\dot{\varsigma }}_{i}\left(t\right)=-\mu \chi \left(\varsigma_{i}\left(t\right)\right)+\rho_{i}. \end{array}$$

We present the following Lyapunov candidate function $F_{3}\left(t\right)=\varsigma_{i}^{2}\left(t\right)/2$ and its time derivative is written as


(24) $${\dot{F}}_{3}(t)=\varsigma_{i}(t)\left(-\mu \chi \left(\varsigma_{i}(t)\right)+\rho_{i}\right).$$

Obviously, the sign of the ς_*i*_(*t*) will affect the positive and negative of Ḟ_3_(*t*). Therefore, we divide ς_*i*_(*t*) into three situations and discussed them in detail one by one.

#### 4.2.1 ς_*i*_(*t*) < 0

In this case, according to the definition of power bounded adaptive function, we have χ(ς_*i*_(*t*)) < 0. Consequently, the following three subcases are provided to guarantee the negative definiteness of Ḟ_3_(*t*).

- Firstly, in the situation of −μχ(ς_*i*_(*t*))+ρ_*i*_>0. On account of ς_*i*_(*t*) < 0 and Equation 24, the time derivative of candidate function Ḟ_3_(*t*) < 0. Therefore, according to the Lyapunov theory, we can summarize that the system (Equation 23) is globally convergent. That is to say, −μχ(ς_*i*_(*t*)) approaches to constant noise ρ_*i*_ over time until −μχ(ς_*i*_(*t*))+ρ_*i*_ = 0. In addition, the convergence performance of model (Equation 23) will be demonstrated in the following text simulation.
- Secondly, in the situation of −μχ(ς_*i*_(*t*))+ρ_*i*_ = 0. Evidently, it can infer that Ḟ_3_(*t*) = 0 and $\varsigma_{i}\left(t\right)=\chi^{-1}\left(\rho_{i}/\mu \right)$. In general, the system (Equation 23) is steady and ς_*i*_(*t*) convergent to a ball surface will be verified again by the following simulation.
- Thirdly, in the situation of −μχ(ς_*i*_(*t*))+ρ_*i*_ < 0. Due to ς_*i*_(*t*) < 0 and −μχ(ς_*i*_(*t*))+ρ_*i*_ < 0, Ḟ_3_>0. It can be readily deduced that the system (Equation 23) diverges and the absolute value of χ(ς_*i*_(*t*)) grows bigger due to the absolute value of ς_*i*_(*t*) turn larger. In light of the power-bounded adaptive activation function's upper χ+ and lower χ− bounds will influent the robustness of the system (Equation 23) in this situation, therefore, we further divide the situation into the following two subcases. When μχ− ≤ ρ_*i*_, there always exists a time instant *t* to transform system (Equation 23) in to case of −μχ(ς_*i*_(*t*))+ρ_*i*_ = 0, which infer the system tend to steady. On the contrary, when μχ−>ρ_*i*_, the system (Equation 23) diverges as time evolves. Consequently, to avoid the divergence of the system, it is necessary to properly adjust the value of scale parameter μ in Equation 23 as well as Equation 13.

#### 4.2.2 ς_*i*_(*t*) = 0

Obviously, in this sence χ(ς_*i*_(*t*)) = 0 and ${\dot{\varsigma }}_{i}\left(t\right)=\rho_{i}$. It manifests that ς_*i*_(*t*)>0 when constant noise ρ_*i*_>0 or ς_*i*_(*t*) < 0 when ρ_*i*_ < 0. Accordingly, ς_*i*_(*t*) only exist as a transient state and the system (Equation 23) is unstable when ρ_*i*_≠0, which indicates the situation will turn back to case in ς_*i*_(*t*) < 0 or ς_*i*_(*t*)>0.

#### 4.2.3 ς_*i*_(*t*)>0

The situation in this part is similar to the situation when ς_*i*_(*t*) < 0, so it is omitted there.

In view of 0 < ρ < μχ+ or μχ− < ρ < 0, $\underset{t\to \infty }{\lim}\varsigma \left(t\right)=\chi^{-1}\left(\rho /\mu \right)$. Next, it can be obtained that $\underset{t\to \infty }{\lim}\overset{{}^{\circ}}{\varsigma }\left(t\right)=0$. Thus, according to the above conditions, at time *t* tending to infinity, $\dot{\varsigma }\left(t\right)=\dot{\epsilon }\left(t\right)+\gamma \varrho \left(\epsilon \left(t\right)\right)=0$. The following equation is derived from Equation 12:


(25) $$\begin{array}{l} \dot{\epsilon }\left(t\right)=-\gamma \varrho \left(\epsilon \left(t\right)\right). \end{array}$$

The derivation of Equation 25 can be obtained from Theorem 1, so the proof is omitted here. Further, in view of 0 < ρ < μχ+ or μχ− < ρ < 0, $\dot{\varsigma }\left(t\right)=-\mu \chi \left(\varsigma \left(t\right)\right)+\rho \leq 0$, that is to say, the NIZND model (Equation 13) for solving the DSSL (Equation 6) globally converges to zero.

The proof is completed.

*Theorem 3:* Beginning with a randomly generated initial position vector ***p***(0), the residual error of the NIZND (Equation 13) model perturbed by the bounded random noise ***ℏ***(*t*) converges to a bounded range, where ***ρ***(*t*) = ***ℏ***(*t*)∈ℝ^*n*^ represents the bounded random noise.

*Proof:* Taking into account the interference of bounded random noise ***ℏ***(*t*), the activation function is set uniformly as a linear activation function, so that the NIZND model (Equation 13) be equivalent to the following equation in this situation as follows:


(26) $$\begin{array}{l} \dot{\epsilon }\left(t\right)=\left(-\gamma -\mu \right)\epsilon \left(t\right)-\gamma \mu \int_{0}^{t}\epsilon \left(\tau \right)\text{d}\tau . \end{array}$$

By defining


$$\begin{array}{l} s_{i}\left(t\right)=\left[\begin{array}{c} \epsilon_{i}\left(t\right)\\ \int_{0}^{t}\epsilon_{i}\left(\tau \right)\text{d}\tau \end{array}\right],V=\left[\begin{array}{cc} -\gamma -\mu & -\gamma \mu \\ 1 & 0 \end{array}\right],w=\left[\begin{array}{c} 1\\ 0 \end{array}\right], \end{array}$$

the Equation 26 can be written as


(27) $$\begin{array}{l} {\dot{s}}_{i}=Vs_{i}\left(t\right)+w\hbar_{i}\left(t\right), \end{array}$$

where ℏ_*i*_(*t*) denoted the *i*th element of the bounded random noise ***ℏ***(*t*). Moreover, it is elicited that:


(28) $$\begin{array}{l} s_{i}\left(t\right)=\exp\left(Vt\right)s_{i}\left(0\right)+\int_{0}^{t}\exp\left(V\left(t-\tau \right)\right)w\hbar_{i}\left(\tau \right)\text{d}\tau . \end{array}$$

In terms of the definition of the triangle inequality, the following inequation is obtained as


(29) $$\begin{array}{l} ||s_{i}\left(t\right)|{|}_{2}\leq ||\exp\left(Vt\right)s_{i}\left(0\right)|{|}_{2}\\ \;+||\int_{0}^{t}\exp\left(V\left(t-\tau \right)\right)w\hbar_{i}\left(\tau \right)\text{d}\tau |{|}_{2}\\ \;\leq ||\exp\left(Vt\right)s_{i}\left(0\right)|{|}_{2}\\ \;+\int_{0}^{t}||\exp\left(V\left(t-\tau \right)\right)w|{|}_{2}|\hbar_{i}\left(\tau \right)|\text{d}\tau . \end{array}$$

To further solve the linear differential equation with higher order constant coefficients (Equation 26), it can be divided into the following three cases according to the parameter Δ = (γ+μ)^2^ − 4*γμ*.

**Case I**: For the case of Δ>0, it can be easily premised that $\Gamma_{1,2}=\left(\left(-\gamma -\mu \right)\pm \sqrt{{\left(\gamma +\mu \right)}^{2}-4\gamma \mu }\right)/2$, from which Γ_1_≠Γ_2_. Thus, it can be gotten that:


$$\begin{array}{c} \exp\left(Vt\right)s_{i}\left(0\right)=\left[\begin{array}{c} \frac{\epsilon_{i}\left(0\right)\left(\Gamma_{1}\exp\left(\Gamma_{1}t\right)-\Gamma_{2}\exp\left(\Gamma_{2}t\right)\right)}{\Gamma_{1}-\Gamma_{2}}\\ \frac{\epsilon_{i}\left(0\right)\left(\exp\left(\Gamma_{1}t\right)-\exp\left(\Gamma_{2}t\right)\right)}{\Gamma_{1}-\Gamma_{2}} \end{array}\right],\\ \exp\left(Vt\right)w=\left[\begin{array}{c} \frac{\left(\Gamma_{1}\exp\left(\Gamma_{1}t\right)-\Gamma_{2}\exp\left(\Gamma_{2}t\right)\right)}{\Gamma_{1}-\Gamma_{2}}\\ \frac{\left(\exp\left(\Gamma_{1}t\right)-\exp\left(\Gamma_{2}t\right)\right)}{\Gamma_{1}-\Gamma_{2}} \end{array}\right], \end{array}$$

where Γ_1_ = −μ, Γ_2_ = −γ, Γ_1_≠Γ_2_. In order to discuss the magnitude of the values of A and B, they are divided into two cases for analysis in detail.

- For the subcase of Γ_1_>Γ_2_, it is naturally acquired that (Γ_1_exp(Γ_1_*t*)−Γ_2_exp(Γ_2_*t*))/(Γ_1_−Γ_2_) < exp(Γ_1_*t*) and (exp(Γ_1_*t*)−exp(Γ_2_*t*))/(Γ_1_−Γ_2_) < exp(Γ_1_*t*)/(Γ_1_−Γ_2_). Thus, it is further obtained that:

$$\begin{array}{c} ||\exp\left(Vt\right)s_{i}\left(t\right)|{|}_{2}\leq \frac{\sqrt{{\left(\gamma -\mu \right)}^{2}+1}}{\gamma -\mu }\exp\left(\Gamma_{1}t\right)|\epsilon_{i}\left(0\right)|,\\ ||\exp\left(Vt\right)w|{|}_{2}\leq \frac{\sqrt{{\left(\gamma -\mu \right)}^{2}+1}}{\gamma -\mu }\exp\left(\Gamma_{1}t\right). \end{array}$$

Then, we could get:

$$\begin{array}{r} \left|\epsilon_{i}(t)\right|\leq {\left\|s_{i}(t)\right\|}_{2}\leq \frac{\sqrt{{\left(\gamma -\mu \right)}^{2}+1}}{\gamma -\mu }\exp\left(\Gamma_{1}t\right)\left|\epsilon_{i}(0)\right|\\ -\frac{\sqrt{{\left(\gamma -\mu \right)}^{2}+1}}{\Gamma_{1}(\gamma -\mu )}\underset{0<\tau <t}{\max}\left|\hbar_{i}(\tau )\right|. \end{array}$$

At last, the upper bound of error can be calculated as follows:

$$\begin{array}{l} \underset{t\to \infty }{\lim}\sup||\epsilon \left(t\right)|{|}_{2}\leq \frac{\psi \sqrt{k\left({\left(\gamma -\mu \right)}^{2}+1\right)}}{\mu \left(\gamma -\mu \right)}. \end{array}$$

- For the subcase of Γ_1_ < Γ_2_, likewise, the proof process is the same as for Γ_1_>Γ_2_. Thus, we can deduce that $\underset{t\to \infty }{\lim}\sup||\epsilon \left(t\right)|{|}_{2}\leq \frac{\psi \sqrt{k\left({\left(\gamma -\mu \right)}^{2}+1\right)}}{\mu \left(\gamma -\mu \right)}$. **Case II**: For the case of Δ = 0, it can be clearly inferred that μ = γ. In view of the above condition, we can know that:

$$\begin{array}{c} \exp\left(Vt\right)s_{i}\left(0\right)=\left[\begin{array}{c} \epsilon_{i}\left(0\right)\Gamma_{1}t\exp\left(\Gamma_{1}t\right)\\ \epsilon_{i}\left(0\right)t\exp\left(\Gamma_{1}t\right) \end{array}\right],\\ \exp\left(Vt\right)w=\left[\begin{array}{c} \Gamma_{1}t\exp\left(\Gamma_{1}t\right)\\ t\exp\left(\Gamma_{1}t\right) \end{array}\right], \end{array}$$

from which Γ_1_ = Γ_2_ = (−γ−μ)/2. According to the proof of Lemma 1 in , $t\sqrt{\Gamma_{1}^{2}+1}\exp\left(\Gamma_{1}t\right)<\upsilon \exp\left(-\delta t\right)$ with υ>0, δ>0.

$$\begin{array}{l} ||\exp\left(Vt\right)w|{|}_{2}=t\sqrt{\Gamma_{1}^{2}+1}\exp\left(\Gamma_{1}t\right)<\upsilon \exp\left(-\delta t\right), \end{array}$$

and

$$\begin{array}{l} |\epsilon_{i}\left(t\right)|\leq ||s_{i}\left(t\right)|{|}_{2}\leq \upsilon \exp\left(-\delta t\right)|\epsilon_{i}\left(0\right)|+\frac{\upsilon }{\delta }\underset{0<\tau <t}{\max}|\hbar_{i}\left(\tau \right)|. \end{array}$$

Finally,

$$\begin{array}{l} \underset{t\to \infty }{\lim}\sup||\epsilon \left(t\right)|{|}_{2}\leq \frac{\psi \upsilon \sqrt{k}}{\delta }, \end{array}$$

where $\psi =\underset{1\leq i\leq k}{\max}\left\{\underset{0\leq \tau \leq t}{\max}|\hbar_{i}\left(\tau \right)|\right\}$. Therefore, perturbed by the bounded random noise ***ℏ***(*t*), the residual errors ***ϵ***(*t*) of the proposed NIZND model (Equation 13) for solving DSSL problem (Equation 6) are bounded. The proof is completed.

## 5 Illustrative simulation experiments

In this section, corresponding localization examples and compare experiments are designed and performed to demonstrate the feasibility, efficiency, and superiority of the proposed NIZND model (Equation 13). Note that all the following simulation experiments are run on a computer with an AMD Ryzen 5 5600H with Radeon Graphics @3.30 GHz CPU, 16-GB memory, NVIDIA GeForce RTXTM 3050 GPU, and Windows 11, 64-bit operating system.

### 5.1 Simulation experiments on AoA

Firstly, five sets of randomly-generated initial states are utilized to solve the DSSL problem (Equation 6) by using the NIZND model (Equation 13), with the corresponding initial conditions picked as follows. We choose the number of base stations to be 8, the coordinates of base stations:


$$\begin{array}{l} S=\left[\begin{array}{cccccccc} -11 & 10 & -11 & 10 & -11 & 10 & -11 & 10\\ -10 & -10 & 10 & 10 & -10 & -10 & 10 & 10\\ -10 & -10 & -10 & -10 & 30 & 30 & 30 & 30 \end{array}\right], \end{array}$$

the five sets of randomly-generated initial states:


$$\begin{array}{l} P\left(0\right)=\left[\begin{array}{ccccc} -5 & -2 & 0.5 & -2 & -5\\ 5 & 3 & 2.5 & -3 & 0\\ 0 & -5 & -0.5 & -2 & -5 \end{array}\right], \end{array}$$

the trajectory position of the target object in 3-D space:


$$\begin{array}{l} p^{*}\left(t\right)=\left[\begin{array}{c} 5\text{cos}\left(5t\right)\\ 5\text{sin}\left(5t\right)\\ 10t \end{array}\right], \end{array}$$

the scalar-oriented and the feedback-oriented activation function:


$$\begin{array}{l} \varrho \left(\epsilon_{i}\right)=20*{\text{Lip}}^{3}\left(\epsilon_{i}\right)+20*{\text{Lip}}^{\frac{1}{3}}\left(\epsilon_{i}\right). \end{array}$$

**Figure 3:**
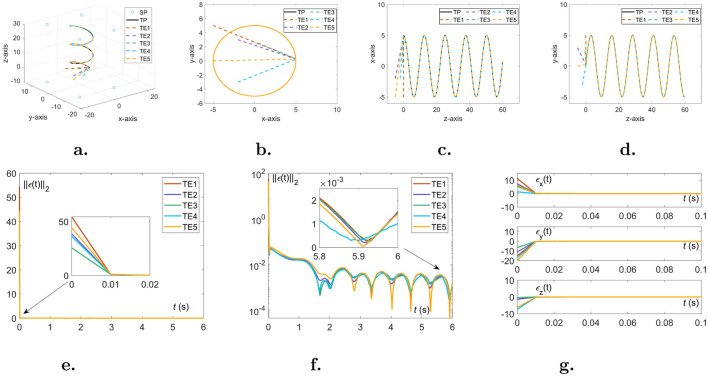
Five sets of unequal initial values are randomly generated to solve the DSSL problem (Equation 6) with the AoA scheme (Equations 1, 2) under noise-free conditions. **(A)** Three-dimensional trajectory map. **(B)** Top view. **(C)** Main view. **(D)** Left view. **(E)** The linear representation of the approximation errors ||***ϵ***(*t*)||_2_. **(F)** The logarithmic representation of the approximation errors ||***ϵ***(*t*)||_2_. **(G)** The components of approximation errors in the x,y,z directions.

Secondly, the purpose of Figure 3 is to demonstrate the validity of Theorem 1 by means of the AoA scheme (Equations 1, 2). Specifically, the simulation results among the five sets of initial values solved by the proposed NIZND model (Equation 13) of AoA scheme (Equations 1, 2) are demonstrated in Figure 3. As shown in Figures 3A–D, a comparison of five different initial values of results reveals that the trajectories of the five sets of random initial values approximately coincide with the theoretical value trajectory. Moreover, it is worth noting that from Figures 3E–G, the performance is evaluated from the linear representation, logarithmic representation of approximation errors, and the components of approximation errors in the x,y,z directions, which prove that the proposed NIZND model (Equation 13) globally converges to zero. Therefore, the proposed NIZND model (Equation 13) can be solved for the DSSL problem (Equation 6) at a certain time by the AoA scheme (Equations 1, 2). Similarly, the results of the correlational analysis are set out in Table 1. The average steady-state residual error and maximum steady-state residual error of the NIZND model (Equation 13) converge to 2.918 × 10^−3^ and 6.402 × 10^−3^ when γ = μ = 3, 8.627 × 10^−3^ and 1.885 × 10^−3^ when γ = μ = 20, which is less than the two residual errors of TGND model (Equation 9) and TZND model (Equation 11) under the same conditions.

**Table 1:** Comparison of the average steady-state residual error (ASSRE) and maximum steady-state residual error (MSSRE) among TGND model, TZND model, and the proposed NIZND model (Equation 13) when solving the DSSL problem (Equation 6) using the AoA scheme (Equations 1, 2).

		**Average steady-state residual error**				**Maximal steady-state residual error**			
**Model**	**Parameters**	**with NF** ^⋆^	**with CN** ^⋆^	**with LN** ^⋆^	**with RN** ^⋆^	**with NF** ^⋆^	**with CN** ^⋆^	**with LN** ^⋆^	**with RN** ^⋆^
TGND	ω = 3	2.325 × 10^0^	2.252 × 10^1^	Infinity	1.084 × 10^1^	3.010 × 10^0^	2.520 × 10^1^	Infinity	1.208 × 10^1^
	ω = 20	3.385 × 10^−1^	2.347 × 10^1^	Infinity	1.166 × 10^1^	4.614 × 10^−1^	2.467 × 10^1^	Infinity	1.248 × 10^1^
TZND	κ = 3	1.784 × 10^−2^	7.879 × 10^0^	Infinity	3.959 × 10^0^	7.809 × 10^−2^	8.654 × 10^0^	Infinity	4.179 × 10^0^
	κ = 20	7.401 × 10^−3^	1.181 × 10^0^	Infinity	5.926 × 10^−1^	2.844 × 10^−2^	1.236 × 10^0^	Infinity	7.181 × 10^−1^
NIZND	γ = 3, μ = 3	2.918 × 10^−3^	1.239 × 10^−2^	3.352 × 10^−2^	1.706 × 10^−2^	6.402 × 10^−3^	2.422 × 10^−2^	3.983 × 10^−2^	3.993 × 10^−2^
	γ = 20, μ = 20	8.267 × 10^−4^	1.145 × 10^−3^	1.472 × 10^−3^	3.267 × 10^−3^	1.885 × 10^−3^	2.736 × 10^−3^	3.116 × 10^−3^	8.667 × 10^−3^

^⋆^NF, CN, LN, and RN indicate noise free, constant noise 8, linear noise 2*t*, and random noise 8 × [0, 1], respectively.

### 5.2 Simulation experiments on TDOA

**Figure 4:**
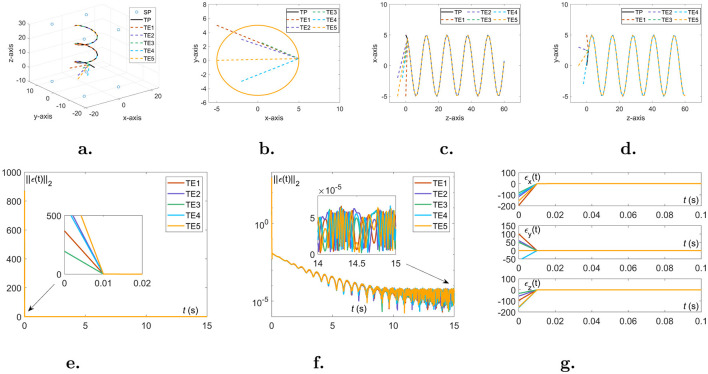
Five sets of unequal initial values are randomly generated to solve the DSSL problem (Equation 6) with the TDOA scheme (Equation 4) under noise-free conditions. **(A)** Three-dimensional trajectory map. **(B)** Top view. **(C)** Main view. **(D)** Left view. **(E)** The linear representation of the approximation errors ||***ϵ***(*t*)||_2_. **(F)** The logarithmic representation of the approximation errors ||***ϵ***(*t*)||_2_. **(G)** The components of approximation errors in the x,y,z directions.

In this part, the experiments on the TDOA scheme 4 shown in Figure 4 also reveal the validity of Theorem 1. For comparison, initial conditions for the TDOA scheme 4 simulation experiments are the same as in the previous section. The corresponding performance comparisons of the proposed NIZND model (Equation 13) are referenced by Figure 4 and Table 2. From the overlap degree in Figures 4A–D, choosing five sets of random initial values, the NIZND model (Equation 13) can gradually converge to a theoretical solution by solving the DSSL problem (Equation 6) with the TDOA scheme (Equation 4) in a noise-free operating environment. Additionally, the results obtained from the preliminary analysis of convergence performance can be seen that the proposed NIZND model (Equation 13) globally converges to zero in Figures 4E–G. Overall, the TDOA scheme (Equation 4) can be solved by the proposed NIZND model (Equation 13) and used to solve the DSSL problem (Equation 6). Further analysis of the data is presented in Table 2. As can be seen from the table above, the first group reported significantly less average steady-state residual error and maximum steady-state residual error than the other three groups under the same model. Moreover, the average steady-state residual error and maximum steady-state residual error for the NIZND model (Equation 13) are 3.917 × 10^−5^ and 7.236 × 10^−5^, respectively, when the coefficients γ = μ = 3; and 3.895 × 10^−4^ and 7.207 × 10^−4^, respectively, when the coefficients γ = μ = 20. Taken together, these results show that the convergence accuracy of the proposed NIZND model (Equation 13) for solving the DSSL problem (Equation 6) under the same conditions is higher than that of the other two models.

**Table 2:** Comparison of the average steady-state residual error (ASSRE) and maximum steady-state residual error (MSSRE) among TGND model, TZND model, and the proposed NIZND model (Equation 13) when solving the DSSL problem (Equation 6) using the TDOA scheme (Equation 4).

		**Average steady-state residual error**				**Maximal steady-state residual error**			
**Model**	**Parameters**	**with NF** ^⋆^	**with CN** ^⋆^	**with LN** ^⋆^	**with RN** ^⋆^	**with NF** ^⋆^	**with CN** ^⋆^	**with LN** ^⋆^	**with RN** ^⋆^
TGND	ω = 3	6.404 × 10^0^	2.019 × 10^1^	Infinity	1.043 × 10^1^	1.037 × 10^1^	2.225 × 10^1^	Infinity	1.541 × 10^1^
	ω = 20	6.193 × 10^−1^	2.017 × 10^1^	Infinity	1.018 × 10^1^	2.024 × 10^0^	2.182 × 10^1^	Infinity	1.483 × 10^1^
TZND	κ = 3	4.726 × 10^−2^	6.418 × 10^0^	Infinity	3.333 × 10^0^	1.174 × 10^−1^	6.719 × 10^0^	Infinity	3.849 × 10^0^
	κ = 20	2.246 × 10^−2^	1.006 × 10^0^	Infinity	5.221 × 10^−1^	6.632 × 10^−2^	1.101 × 10^0^	Infinity	7.771 × 10^−1^
NIZND	γ = 3, μ = 3	3.917 × 10^−5^	5.636 × 10^−3^	2.780 × 10^−2^	1.752 × 10^−2^	7.236 × 10^−5^	1.951 × 10^−2^	3.350 × 10^−2^	3.506 × 10^−2^
	γ = 20, μ = 20	3.895 × 10^−4^	4.241 × 10^−4^	7.759 × 10^−4^	2.607 × 10^−3^	7.207 × 10^−4^	8.047 × 10^−4^	1.557 × 10^−3^	7.007 × 10^−3^

^⋆^NF, CN, LN, and RN indicate noise free, constant noise 8, linear noise 2*t*, and random noise 8 × [0, 1], respectively.

### 5.3 Performance under noise perturbed

**Figure 5:**
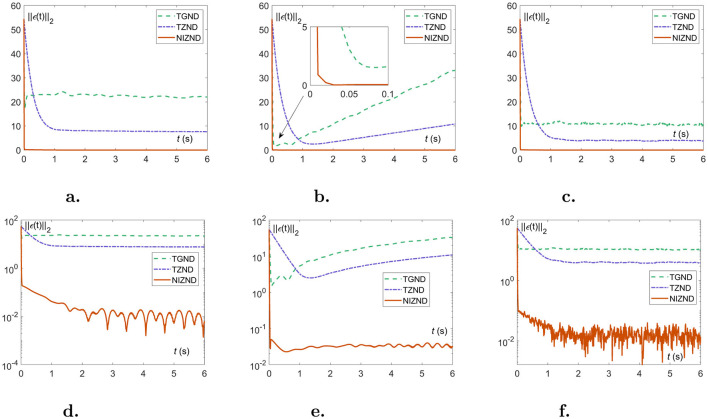
The approximation errors of comparisons among NIZND (Equation 13), TZND (Equation 11) and TGND (Equation 9) models based on AoA scheme under various of noises. **(A)** The linear representation of the approximation error ||***ϵ***(*t*)||_2_ under constant noise. **(B)** The linear representation of the approximation error ||***ϵ***(*t*)||_2_ under linear noise. **(C)** The linear representation of the approximation error ||***ϵ***(*t*)||_2_ under random noise. **(D)** The logarithmic representation of the approximation error ||***ϵ***(*t*)||_2_ under constant noise. **(E)** The logarithmic representation of the approximation error ||***ϵ***(*t*)||_2_ under linear noise. **(F)** The logarithmic representation of the approximation error ||***ϵ***(*t*)||_2_ under random noise.

**Figure 6:**
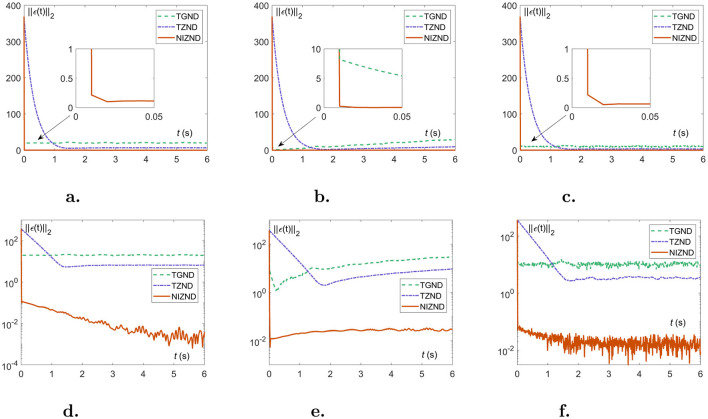
The approximation errors of comparisons among NIZND (Equation 13), TZND (Eqution 11) and TGND (Equation 9) models based on TDOA scheme under various of noises. **(A)** The linear representation of the approximation error ||***ϵ***(*t*)||_2_ under constant noise. **(B)** The linear representation of the approximation error ||***ϵ***(*t*)||_2_ under linear noise. **(C)** The linear representation of the approximation error ||***ϵ***(*t*)||_2_ under random noise. **(D)** The logarithmic representation of the approximation error ||***ϵ***(*t*)||_2_ under constant noise. **(E)** The logarithmic representation of the approximation error ||***ϵ***(*t*)||_2_ under linear noise. **(F)** The logarithmic representation of the approximation error ||***ϵ***(*t*)||_2_ under random noise.

In this part, the approximation errors of two localization schemes are presented in the form of residual plots and numerical tables to show the experimental results of solving the DSSL problem (Equation 6) under various noisy environments, i.e., constant noise ***ρ***(*t*) = 8, linear noise ***ρ***(*t*) = 2*t*, and random noise ***ρ***(*t*)∈8 × [0, 1]. As a comparison, the experimental results of AoA scheme (Equations 1, 2) and TDOA scheme 4 are generated by three models, i.e., TGND model (9), TZND model (11), and the proposed NIZND model (13). The corresponding localization results are shown in Figures 5, 6 and Tables 1, 2. Figures 5, 6 demonstrate the approximation errors ||***ϵ***(*t*)||_2_ of AoA and TDOA scheme under three kinds of noises with parameters ω = κ = γ = μ = 3, respectively.

From the visualization results in Figures 5A, D, it can be seen that the proposed NIZND model (Equation 13) converges promptly to 10^−2^ under constant noises, at which point the proposed NIZND model (13) yields better convergence accuracy than the TGND (Equation 9) and TZND models (Equation 11). At the same time, Figures 5B, E illustrates the proposed NIZND model (Equation 13) converges smoothly to 10^−2^ under the linear noise, while those of the TGND (Equation 9) and TZND models (Equation 11) are of divergence. As Table 1 depicted, the average steady-state residual error and maximum steady-state residual error of the TGND (Equation 9) and TZND (Equation 11) models converge to infinity. Considering the case of linear noises in Figures 5C, F, we can see that the error of the proposed NIZND model (13) resulted in the lowest value of the number. Specifically, Table 1 also shows the same results.

Likewise, the experimental results for the TDOA scheme 4 have a similar trend to the experimental results for the AoA scheme (Equations 1, 2). It is worth mentioning that Figures 6B, E and Table 2 can demonstrate the convergence of the TGND model (Equation 9) and the TZND model (Equation 11) for solving the DSSL problem (Equation 6), which is undoubtedly a great challenge for dynamic localization from the real environment. Under noises situation, combing with the visualization results (Equation 6), both the (Equation 9) and the TZND model (Equation 11) are worse than that of the proposed NIZND model (Equation 13). Of the Table 2, the average steady-state residual error and maximum steady-state residual error of the proposed NIZND model (Equation 13) are also less than those of the other two comparison models. Furthermore, the larger the coefficients γ, μ of the proposed NIZND model (Equation 13), the higher its convergence accuracy under noisy conditions, which indicates the strong robustness of the proposed model (Equation 13).

### 5.4 Application to the robotic manipulator

**Figure 7:**
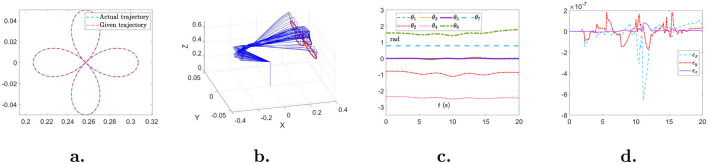
Simulation results of trajectory tracking scheme (Equation 30) solved by the proposed NIZND model (Equation 13). **(A)** The actual trajectory and given trajectory. **(B)** The movement of the robotic manipulator. **(C)** Joint angle. **(D)** Tracking error.

To further demonstrate the effectiveness and robustness of the proposed NIZND model (Equation 13), a simulation is conducted on a robotic manipulator employing NIZND model (Equation 13) for achieving precise trajectory tracking. The trajectory tracking of a robotic manipulator is to obtain the joint angle ***θ***(*t*) for the desired trajectory ***r***_d_(*t*) at each time instant. Specifically, the trajectory tracking of a robotic manipulator is achieved by solving the following equation:


(30) $$\begin{array}{l} J\left(\theta \left(t\right)\right)\dot{\theta }\left(t\right)={\dot{r}}_{\text{d}}\left(t\right), \end{array}$$

where *J*(***θ***(*t*)) represents the Jacobian matrix and $\dot{\theta }\left(t\right)$ is the joint velocity. Then, the proposed NIZND model (Equation 13) is employed to obtain the solution to trajectory tracking scheme (Equation 30) under noise perturbation. Simulation results are shown in Figure 7. Figure 7A depicts the actual trajectory and given trajectory of the robotic manipulator. The movement process is displayed in Figure 7B. Figures 7C, D illustrate the joint angle and tracking error during the tracking task. Solved by NIZND model (Equation 13), the trajectory tracking task is successfully accomplished with minor tracking error, demonstrating the effectiveness and robustness of the proposed NIZND model (Equation 13).

### 5.5 Summary

In summary, the proposed NIZND model (Equation 13) demonstrates superiority over the three models. Moreover, these comparative results suggest an association between convergence performance and the coefficients taken. In the above experiments, by comparing the performance differences between the models under the same noise conditions, it is clear that the proposed NIZND model (Equation 13) is characterized by high convergence accuracy and strong robustness. In general, the proposed NIZND model (Equation 13) has a higher convergence accuracy in the noise-free condition. In addition, its strong robustness enables it to maintain good convergence performance under the three noise conditions. In contrast, the robustness is enhanced by increasing the values of the two coefficients of the proposed NIZND model (Equation 13), which can be adapted to complicated noise disturbances in realistic environments.

## 6 Conclusion

In this paper, a novel noise-immune zeroing neural dynamics (NIZND) model has been proposed for solving the dynamic signal source localization tracking (DSSL) and robotic trajectory tracking problem. Additionally, taking into account the effect of noise in real scenes, four theorems, and the corresponding proof process are presented. Specifically, the results of the proposed theromes are shown that the proposed NIZND model process global convergence and enhances robustness. Then, the superiority of the model in solving the DSSL problem was verified by computer simulations and experiments compared to other models. Additionally, it has been applied in a robotic manipulator to further demonstrate the effectiveness and robustness of the proposed NIZND model. Finally, it is worth mentioning that possible future investigations will optimize the proposed NIZND model to better cope with the problems posed by realistic DSSL.

## Data Availability

The original contributions presented in the study are included in the article/supplementary material, further inquiries can be directed to the corresponding author.
